# Work-Related Shoulder Pain Among Saudi Orthopedic Surgeons: A Cross-Sectional Study

**DOI:** 10.7759/cureus.48023

**Published:** 2023-10-31

**Authors:** Ahmed AlHussain, Nouf A Almagushi, Mohammad S Almosa, Sultan N Alotaibi, Khalid AlHarbi, Abdulelah M Alharbi, Halah Al Shabraqi, Fay Alowid

**Affiliations:** 1 Orthopaedic Surgery, King Abdulaziz Medical City, Riyadh, SAU; 2 College of Medicine, King Saud Bin Abdulaziz University for Health Sciences, Riyadh, SAU; 3 Medicine, King Saud Bin Abdulaziz University for Health Sciences, Riyadh, SAU; 4 Orthopedic Surgery, King Saud University, Riyadh, SAU; 5 Orthopedics, Imam Mohammad Ibn Saud Islamic University, Riyadh, SAU

**Keywords:** pain score questionnaire, orthopedic surgeon, spadi score, shoulder injuries, orthopaedic surgery

## Abstract

Introduction: Shoulder pain is one of the most frequently reported musculoskeletal conditions that approximately 6.9 to 26% of people experience. Numerous etiologies have been linked to causing shoulder pain, with the most frequent one being rotator cuff tendinopathy. Work-related musculoskeletal pain is prevalent in the medical field in general, but orthopaedic surgeons account for the majority of cases overall. The pain experienced is usually caused by many attributing factors that all relate to either the long hours and physically intensive procedures or the unnatural positions orthopaedic surgeons are put in during their surgeries. This study seeks to shed light on the prevalence, contributing factors, and effect of the complications of shoulder pain among orthopaedic surgeons, a topic unexpectedly understudied, particularly in Saudi Arabia.

Methods: A survey has been developed to ask orthopaedic surgeons at various levels of their careers. The study was conducted in Saudi Arabia, Riyadh. It was a multi-centered study that included both governmental and private hospitals in Riyadh. The collected data included age, gender, BMI, sub-specialty, position, number of surgeries performed per week, and the average time per surgery. In our survey, we used the Shoulder Pain and Disability Index (SPADI) to assess shoulder pain. Ethical approval was obtained for this study by the Institutional Review Board of King Abdullah International Medical Research Center, Ministry of National Guard Health Affairs, Riyadh, Kingdom of Saudi Arabia (IRB/1484/23).

Results: Fifty orthopaedic surgeons participated in this study most of whom were male (88%) and fell within the age range of 31-40 years (36%). Regarding their positions, a significant proportion were consultants (54%). In terms of workload, approximately (38%) of the surgeons performed three to four surgeries per week. As for the duration of surgeries, almost half of the participants spent between three and five hours per surgery (48%). When it comes to experiencing pain or difficulties during shoulder activities, a considerable number reported occasional occurrences (36%). Moreover, a substantial majority did not have a history of specific shoulder-related disorders, as (88%) of the participants had none of the mentioned conditions, such as shoulder trauma or disorders like adhesive capsulitis and impingement. Most participants reported difficulties in performing daily activities due to their work-related shoulder pain. Mild pain was the most common reported severity level in all assessed activities.

Conclusion: The present study showed that orthopaedic surgeons in Riyadh, Saudi Arabia, occasionally experience shoulder pain from their jobs. Most of our sample stated that mild shoulder discomfort made it difficult to conduct daily tasks. This study is limited by a relatively low response rate, which may be attributed to the demanding nature of orthopaedic surgery. To promote health among caregivers throughout the kingdom, more studies should be conducted about shoulder pain.

## Introduction

Shoulder pain is one of the most frequently reported musculoskeletal conditions that approximately 6.9 to 26% of people experience [1]. It is deemed to be the third most prevalent musculoskeletal complaint presented to primary healthcare clinics [2,3]. Various etiology have been implicated in causing shoulder pain, with the most frequent one being rotator cuff tendinopathy. Additionally, the pain might originate from the acromioclavicular joint, glenohumeral joint, the neck, and the soft tissue surrounding the shoulder [4].

Surgical literature predominantly focuses on patient health, which is of utmost value. However, the literature regarding surgeons' health is considerably deficient. Surgeons are exposed to a variety of risks in their workplace and practice. Work-related musculoskeletal pain is prevalent in the medical field in general, but orthopaedic surgeons carry the lion's share [4-6]. Orthopaedic surgery is a substantially demanding specialty, both physically and mentally. It was reported that orthopaedic surgeons are more prone to physical injury compared to general surgeons, with shoulder and lower back injuries being the most common complaints [6]. The pain experienced is usually caused by many attributing factors that all relate to either the long hours and physically intensive procedures or the unnatural positions orthopaedic surgeons are put in during their surgeries [7-8]. For instance, the awkward elevation of the arm, prolonged flexing of the neck, and the extended hours of standing in the operating room all make the basis of pain experienced by an orthopaedic surgeon. Additionally, orthopaedic surgeons with underlying risk factors such as advanced age, multiple years of practice, sedentary lifestyle, and obesity are more predisposed to develop the aforementioned shoulder injuries [9].

Remarkably, there is no correlation between year of practice, gender, age, or the number of operations and the increased likelihood of musculoskeletal injury; however, it is still a contested topic [9-11]. In contrast to which hand the surgeon prefers, it showed that right-handed surgeons had an increased rate of injury compared to their left-handed counterparts [9].

This study seeks to delve deep and shed light on the prevalence of shoulder pain among orthopaedic surgeons, a topic unexpectedly understudied, particularly in Saudi Arabia. We also seek to understand the factors and characteristics contributing to shoulder pain, as well as the effect of these complications on the daily practice of orthopaedic surgeons both inside and outside of the operation room. The primary objective of this study is to determine the prevalence of shoulder pain in orthopaedic surgeons, which will give us a good jumping point to try to prevent it.

## Materials and methods

The process of designing the questionnaire involved the creation of a meticulously developed electronic survey instrument. The survey encompassed inquiries pertaining to participants' demographic characteristics, including age, gender, approximate body mass index (BMI), and professional position (Resident, Fellow, Registrar, or Consultant). Furthermore, information regarding their specialty, weekly surgical caseload, and average surgery duration was collected. The sampling technique in this study was a convenience non-probability sampling technique. We included both who are in private and governmental hospitals. To assess participants' shoulder pain, the survey incorporated the utilization of the Shoulder Pain and Disability Index (SPADI) questionnaire. This particular questionnaire has been previously employed in a multitude of existing literature and underwent careful revision to ensure better alignment with the objectives of our study. It is noteworthy that no significant modifications were made that could potentially compromise the validity or reliability of the survey instrument. Moreover, the administration of the questionnaire was carried out in the English language. Ethical approval was obtained for this study by the Institutional Review Board of King Abdullah International Medical Research Center, Ministry of National Guard Health Affairs, Riyadh, Kingdom of Saudi Arabia (IRB/1484/23).

The study was conducted in the capital city of Saudi Arabia, Riyadh, and consisted of 50 physicians across multiple hospitals in Riyadh. The participating hospitals in Riyadh varied in size, specialization, and patient population, contributing to the diversity of the sample. Our study consisted of both male and female orthopaedic surgeons and in all different levels of orthopaedic surgical training.

Orthopaedic surgeons' data were collected through an electronic survey that was sent to them through social channels, and variables such as age and gender, approximate BMI and position (Resident, Fellow, Registrar, or Consultant), sub-speciality, number of surgeries performed per week, and the average time per surgery were collected. It was then organized into an Excel sheet.

The Shoulder Pain and Disability Index (SPADI) is a widely used questionnaire that assesses the severity of shoulder pain and disability. It consists of 13 items, which are divided into two subscales: the Pain subscale and the Disability subscale. The pain scale consisted of five questions while the disability scale consisted of eight questions. The questions of the pain scale were summed resulting in a numerical score variable. A numerical percentage variable was calculated from the outcome numerical score variable. The questions of the disability scale as well as all the questions of both scales were summed by the same method, resulting in a numerical percentage variable for disability and a numerical percentage variable for all the questions. The SPADI is considered a reliable and valid measure for evaluating shoulder pain and disability and is frequently used in clinical and research settings [12].

The statistical analysis was done by RStudio (R version 4.3.0; R Foundation for Statistical Computing, Vienna, Austria). The categorical sociodemographic data were presented as frequencies and percentages. To assess the risk factors for high scores of pain, disability, and overall SPADI, we constructed multivariable generalized linear models using each of these scores in a separate model. The following variables were incorporated in the initial multivariable model: gender, age, BMI, position, specialty, number of surgeries performed per week, the average time per surgery, experiencing pain or difficulties when performing shoulder activities and having selected conditions. Subsequently, we implemented a bootstrap procedure to assess the variability of model selection using a backward stepwise algorithm in order to select robust independent variables. A 50-sample bootstrap procedure was performed, and the results of the refitted models containing the finally included variables were demonstrated in relevant tables. The results of the regression analysis were demonstrated as beta coefficients and 95% confidence intervals (95%CIs). Statistical significance was considered at p < 0.05.

## Results

Among the participants, the majority were male (88%) and fell within the age range of 31-40 years (36%). Regarding their positions, a significant proportion were consultants (54%), while most of them specialized in General Orthopaedic Surgery (42%) followed by paediatric orthopaedic surgery (18%). On the other hand only 2% of the participants specialized in sports or arthroplasty. In terms of workload, approximately 38% of the surgeons performed three to four surgeries per week. As for the duration of surgeries, almost half of the participants spent between three and five hours per surgery (48%). When it comes to experiencing pain or difficulties during shoulder activities, a considerable number reported occasional occurrences (36%). Moreover, a substantial majority did not have a history of specific shoulder-related disorders, as 88% of the participants had none of the mentioned conditions, such as shoulder trauma or disorders like adhesive capsulitis and impingement (Table 1). 

**Table 1:** Sociodemographic characteristics of participants (n=50)

Parameter	Category	N	%
Gender	Male	44	88%
	Female	6	12%
Age	20-30	15	30%
	31-40	18	36%
	41-50	9	18%
	More than 50	8	16%
Approximate BMI	18.5-25	19	38%
	26-30	18	36%
	31-35	10	20%
	More than 35	3	6%
Position	Resident	17	34%
	Fellow	2	4%
	Registrar	4	8%
	Consultant	27	54%
Speciality	Spine	4	8%
	Upper Extremity	6	12%
	Sports	2	4%
	Trauma	3	6%
	Oncology	3	6%
	Arthroplasty	2	4%
	General Orthopedic Surgery	21	42%
	Pediatrics	9	18%
Number of surgeries performed per week	2 or Less	7	14%
	3-4	19	38%
	5-6	17	34%
	More than 6	7	14%
The average time per surgery	Less than 3 hours	20	40%
	Between 3 and 5 hours	24	48%
	Between 5 and 7 hours	5	10%
	More than 7 hours	1	2%
Pain or difficulties when performing the shoulder activities	Never	16	32%
	Rarely	18	36%
	Most of the time	15	30%
	Always	1	2%
Do you have any of the following ?	None	44	88%
	History of Spinal or Shoulder trauma (including fractures)	1	2%
	History of shoulder disorders (Adhesive capsulitis, Impingement, etc .. )	4	8%
	History of stroke	1	2%

**Figure 1:**
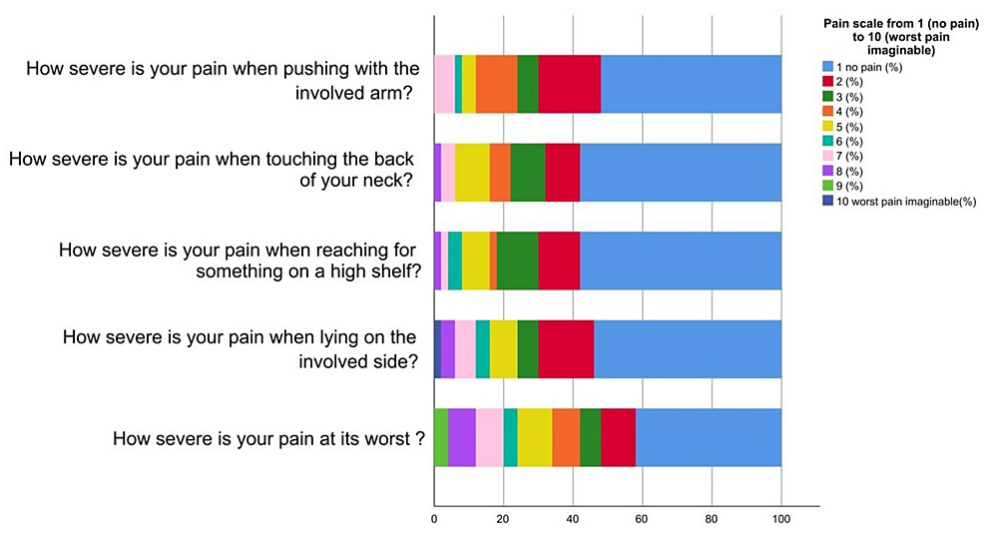
Proportions of participants’ ratings of pain for the items on the pain scale

**Figure 2:**
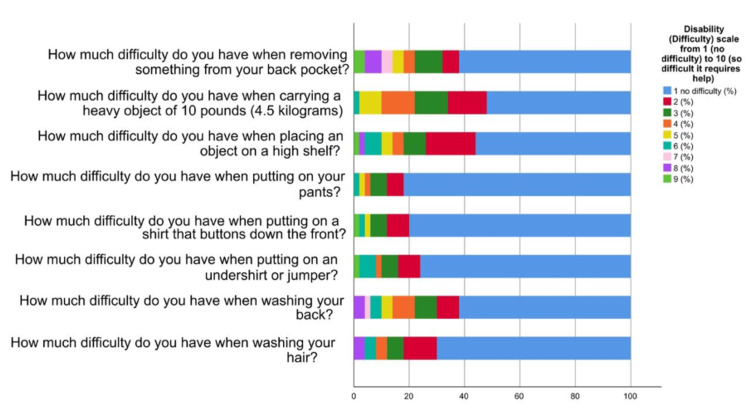
Proportions of participants’ ratings of the items of the disability scale

Figure 1 represents that the majority of orthopaedic surgeons experienced mild pain at its worst (42%) and when lying on the involved side (54%). In addition, the most prevalent pain severity when reaching for something on a high shelf 29 (58%) and touching the back of the neck 29 (58%) was also mild. In terms of pushing with the involved arm, the majority reported mild pain 26 (52%). Overall, mild pain was the most common severity level in all assessed activities. Based on Figure 2, the study reveals that the majority of orthopaedic surgeons reported mild difficulty in performing various daily activities due to their work-related shoulder pain. Activities like washing hair 35 (70%), washing back 31 (62%), putting on an undershirt or jumper 38 (76%), putting on a shirt with buttons 40 (80%), putting on pants 41 (82%), placing an object on a high shelf 28 (56%) and carrying a heavy object of 10 pounds (4.5 kilograms) 26 (52%) were the ones with the highest prevalence of mild difficulty. More than half of the participants had mild difficulty when removing something from their back pocket 31 (62%).

Experiences of pain or difficulties during shoulder activities displayed notable correlations. Surgeons who reported rare occurrences of such pain demonstrated higher pain scores (β = 10.3, p = 0.012), with an even stronger association among those experiencing pain most of the time (β = 37.0, p < 0.001). Among the medical history variables, a history of stroke was significantly linked to elevated pain scores (β = 43.2, p < 0.001), while histories of spinal or shoulder trauma and shoulder disorders did not exhibit statistically significant associations (Table 2).

**Table 2:** Risk factors for high pain scale scores

Characteristic	Beta	95% CI	p-value
The average time per surgery			
Less than three hours	Reference	Reference	
Between three and five hours	3.81	-3.06, 10.7	0.283
Between five and seven hours	-6.44	-17.8, 4.92	0.273
More than seven hours	13.7	-12.9, 40.3	0.318
Have you experienced pain or difficulties when performing any above-the-shoulder activities (e.g., changing bulbs, weight lifting, etc.)?			
Never	Reference	Reference	
Rarely	10.3	2.63, 18.0	0.012
Most of the time	37.0	28.8, 45.1	<0.001
Do you have any of the following?			
None	Reference	Reference	
History of spinal or shoulder trauma (including fractures)	-9.80	-32.7, 13.1	0.407
History of shoulder disorders (adhesive capsulitis, impingement, etc.)	-2.54	-16.3, 11.3	0.720
History of stroke	43.2	20.4, 66.0	<0.001

Regarding age, surgeons aged 31-40 exhibited significantly higher disability scores (β = 25.4, p = 0.037), and a similar trend was observed for those aged over 50 years (β = 29.4, p = 0.029). The professional position also played a role, with fellows displaying significantly lower disability scores (β = -31.2, p = 0.031) compared to residents. Additionally, the number of surgeries performed per week showed significance, with surgeons performing more than six surgeries per week having higher disability scores (β = 13.8, p = 0.048) compared to those performing two or fewer surgeries. Experiencing pain or difficulties during shoulder activities was a substantial contributor, as surgeons who encountered such issues most of the time reported significantly higher disability scores (β = 18.4, p < 0.001). Notably, a history of spinal or shoulder trauma was associated with decreased disability scores (β = -24.8, p = 0.040), while histories of shoulder disorders and stroke did not exhibit statistically significant associations (Table 3). 

**Table 3:** Risk factors for high disability scale scores

Characteristic	Beta	95% CI	p-value
Age (Years)			
20-30	Reference	Reference	
31-40	25.4	2.48, 48.3	0.037
41-50	22.4	-2.49, 47.2	0.087
More than 50	29.4	4.08, 54.8	0.029
Position			
Resident	Reference	Reference	
Fellow	-31.2	-58.4, -4.02	0.031
Registrar	-22.2	-47.8, 3.45	0.099
Consultant	-22.1	-45.8, 1.53	0.076
Number of surgeries performed per week			
Two or Less	Reference	Reference	
Three to four	9.15	-2.06, 20.4	0.119
Five to six	4.81	-6.19, 15.8	0.398
More than six	13.8	0.61, 27.0	0.048
Have you experienced pain or difficulties when performing any above-the-shoulder activities (e.g., changing bulbs, weight lifting, etc.)?			
Never	Reference	Reference	
Rarely	-1.56	-9.91, 6.79	0.717
Most of the time	18.4	10.2, 26.6	<0.001
Always	-10.9	-45.6, 23.7	0.540
Do you have any of the following?			
None	Reference	Reference	
History of spinal or shoulder trauma (including fractures)	-24.8	-47.6, -1.98	0.040
History of shoulder disorders (adhesive capsulitis, impingement, etc .. )	-13.6	-27.1, -0.05	0.057
History of stroke	14.0	-10.6, 38.7	0.273

In terms of professional position, fellows exhibited a significant negative association with SPADI scores (β = -25.9, p = 0.037), indicating lower scores compared to residents. Among the activities of shoulders, surgeons experiencing pain or difficulties most of the time reported significantly higher SPADI scores (β = 26.1, p < 0.001). Variables without statistically significant associations (p ≥ 0.05) include age and having a history of stroke, spinal or shoulder trauma, and history of shoulder disorders (Table 4).

**Table 4:** Risk factors for high SPADI scale scores SPADI: Shoulder Pain and Disability Index

Characteristic	Beta	95% CI	p-value
Age			
20-30	Reference	Reference	
31-40	19.5	-0.48, 39.5	0.063
41-50	14.5	-7.20, 36.2	0.199
More than 50	21.2	-1.35, 43.7	0.073
Position			
Resident	Reference	Reference	
Fellow	-25.9	-49.3, -2.49	0.037
Registrar	-14.3	-36.5, 7.90	0.214
Consultant	-16.0	-36.5, 4.46	0.134
Have you experienced pain or difficulties when performing any above-the-shoulder activities (e.g., changing bulbs, weight lifting, etc.)?			
Never	Reference	Reference	
Rarely	5.63	-1.37, 12.6	0.123
Most of the time	26.1	18.7, 33.6	<0.001
Always	-3.92	-34.7, 26.8	0.804
Do you have any of the following?			
None	Reference	Reference	
History of spinal or shoulder trauma (including fractures)	-18.0	-38.8, 2.85	0.099
History of shoulder disorders (adhesive capsulitis, impingement, etc.)	-8.39	-20.7, 3.89	0.189
History of stroke	21.2	0.14, 42.4	0.056

## Discussion

The postures that orthopaedic surgeons must assume in order to perform their duties are ergonomically challenging. In addition, orthopaedic surgery typically requires significant physical exertion to perform and execute complex surgical techniques [13]. These postures have been recognized as risk factors for musculoskeletal disorders for them [14]. Despite the various risks that orthopaedic surgeons are exposed to in their practice and workplace, there is currently a significant dearth of research on their health. To address this gap, our study aimed to determine the prevalence of work-related shoulder pain among Saudi orthopaedic surgeons and assess the associated risk factors.

While there is ample literature on occupation-related musculoskeletal (MSK) pain and its prevalence in various professions, there has been a notable lack of research on orthopaedic surgeons and their experience of shoulder and upper neck pain in particular. Our findings indicate that a majority of orthopedic surgeons experience mild difficulty in performing daily activities due to work-related shoulder pain, with significant differences in pain scale scores observed between genders. It is noteworthy that most surgeons perform only between three and four surgeries per week. Al Mulhim et al.'s findings regarding that were comparable to ours [15]. Our study also revealed that female participants had higher median scores than their male counterparts. There could be several possible explanations for this gender difference in prevalence, such as varying exposure to risk factors between male and female workers, or the possibility that women have a lower pain threshold [16]. Another potential explanation for this contrast is that a considerable proportion of female patients may be suffering from non-specific pain, which is often linked to psychological or psychosocial factors [17,18]. Additionally, they might be more prone to express pain and symptoms [19]. On the other hand, a study discussing the prevalence of musculoskeletal disorders in trauma orthopedic surgeons found that there is no association between gender and the prevalence of disorders or regions involved [9]. Digging deeper into the association between pain and its influence on the daily activities of surgeons, our results showed that various everyday actions such as buttoning clothing, reaching for far objects, and carrying heavy objects lead to mild shoulder pain. This shoulder discomfort could be the result or the spreading of a misdiagnosed upper neck pain that radiates to the shoulders, which mimics its effect. A study with a similar sample size to ours showed that most orthopaedic surgeons had mild neck pain that caused a reduction in the comfort of performing surgeries, resulting in either missing days of work or considering the option of early retirement. Similarly to our findings, some participants reported that their pain has caused an accelerated decrease in the quality of their life as a result of pain during daily activities [20]. Speciality-wise, no significant association between the disability score index, pain scale score, and subspecialty of the practicing surgeon was found. A study done in Saudi Arabia with 179 orthopaedic surgeons found that there was no correlation between MSK pain at different body sites and the subspecialty of surgeon, only age, years of experience, smoking, and twisting or bending for better access showed significant association [21].

Orthopedic surgeons and the general population may differ in their experiences of pain. The general population faces pain due to various medical conditions, injuries, or chronic illnesses while orthopedic surgeons' work often involves physically demanding tasks which can potentially lead to musculoskeletal pain and discomfort. Research has indicated that orthopedic surgeons may have a higher prevalence of work-related musculoskeletal pain compared to the general population due to the nature of their profession [22]. An important note to keep in mind is that pain perception and tolerance do vary among individuals, and factors such as personal experiences, genetics, and psychological factors may influence an individual's response to pain [22]. Further research and specific studies on pain perception and orthopedic surgeons would give a more clear understanding of this topic.

Our study is intended to shed light on musculoskeletal complaints among healthcare workers, particularly orthopaedic surgeons, and we anticipate that our results will inform ergonomic considerations that can enhance their working conditions. Ultimately, such improvements may have a positive impact on the quality of care provided to patients. Regarding the limitations of our study, the sample is limited to orthopedic surgeons who reside in Riyadh, Saudi Arabia, which makes the sample size restricted and does not provide a broader scope on the relation between occupation-induced shoulder pain and the workload of different working hospitals. Furthermore, the demographics of our sample showed discrepancies in the number of responses between males and females leading to the incapability of finding an association between gender and shoulder pain. In addition, since the results are based on self-reported answers, this increases the incidence of recall bias. Future research could investigate the association of shoulder pain and its risk factors, along with the intraoperative effect on surgeons.

## Conclusions

In summary, our study shows that Saudi orthopaedic doctors in Riyadh, Saudi Arabia, occasionally experience shoulder pain from their jobs. The majority of orthopaedic surgeons in our sample stated that mild shoulder discomfort made it difficult to conduct daily tasks; nevertheless, female participants had higher pain median scores than male participants. The pain scale score and the disability score index did not correlate with the practicing surgeon's subspecialty. In order to promote health among caregivers throughout the kingdom, more studies should be conducted on the subject of shoulder pain.
